# To use or not use lisdexamfetamine in pregnancy and breastfeeding: a case report highlighting ADHD management and maternal–fetal outcomes

**DOI:** 10.1177/26334941261438664

**Published:** 2026-04-05

**Authors:** Tammy H. Tran, Marissa L. Beal, Melissa F. Free, Ritika Baweja

**Affiliations:** Pennsylvania State University College of Medicine, Milton S. Hershey Medical Center, 700 HMC Crescent Road, Hershey, PA 17033, USA; Department of Psychiatry and Behavioral Health, Penn State Milton S. Hershey Medical Center, Hershey, PA, USA; Department of Psychiatry and Behavioral Health, Penn State Milton S. Hershey Medical Center, Hershey, PA, USA; Department of Psychiatry and Behavioral Health, Penn State Milton S. Hershey Medical Center, Hershey, PA, USA

**Keywords:** attention-deficit/hyperactivity disorder, clinical pharmacology, perinatal stimulant use, reproductive psychopharmacology

## Abstract

Attention-deficit hyperactivity disorder (ADHD) is a developmental disorder characterized by inattentiveness, hyperactivity, and impulsivity, often treated with amphetamine and methylphenidate agents. There is limited published data to provide established guidelines for stimulant use during pregnancy, and even less data on particular medications. This case study reports maternal–fetal outcomes with lisdexamfetamine (Vyvanse) use during pregnancy and breastfeeding. The patient who received this medication during her second pregnancy experienced significant improvement in her mental well-being compared to her first pregnancy, despite a similar pregnancy course and fetal outcomes, including preterm delivery, neonatal intensive care unit admission, cleft palate, and micrognathia in both pregnancies. In addition, this case demonstrates one instance of successful breastfeeding with maternal use of lisdexamfetamine with no observed side effects in the infant. This report highlights the importance of managing ADHD during pregnancy and the postpartum period and offers an example of breastfeeding without significant side effects or concerns.

## Introduction

Attention-deficit hyperactivity disorder (ADHD) is a neurodevelopmental childhood disorder that often persists from childhood to adulthood with symptoms of inattentiveness, hyperactivity, and/or impulsivity.^1^ The treatment for ADHD consists of pharmacological or nonpharmacological approaches. Pharmacological options include stimulant medications, such as amphetamines and methylphenidate, as well as nonstimulants like atomoxetine, guanfacine, clonidine, bupropion, and viloxazine. Nonpharmacological management includes cognitive behavioral therapy and behavioral skills development. Current expert consensus and available clinical practice recommendations for adults suggest psychostimulants (such as amphetamines and methylphenidate) as first-line pharmacological treatments, with nonstimulant agents considered for those who do not tolerate or have contraindications to stimulants.^2^ These recommendations are largely extrapolated from the more established pediatric ADHD guidelines and adult clinical consensus, underscoring the need for pregnancy-specific guidance. Data are limited regarding stimulant use during the perinatal period in humans without clear guidelines for use.^3,4^

Appropriate treatment of ADHD in the perinatal period may help prevent worsening of occupational, interpersonal, and daily life impairments and reduce psychosocial stress in pregnancy. Current literature shows that inattentive symptoms during pregnancy are the strongest predictors of daily-life impairments, with difficulties in tasks such as completing household chores, caring for other family members, and managing driving-related responsibilities.^5^ Impulsivity may lead to poor financial management and more difficult interpersonal interactions during pregnancy. These negative impacts of ADHD highlight the importance of effective management and treatment to minimize additional stress. Evidence has also shown that women who experience high levels of psychosocial stress during pregnancy are at increased risk for obstetric complications, including preterm labor, preterm delivery, early onset of spontaneous labor, and low birth weight.^6^ In addition, Baker et al.^7^ demonstrate that discontinuation of psychostimulant treatment during pregnancy adversely affected maternal mental health, with a clinically significant increase in Edinburgh Postnatal Depression Scale (EPDS) and significant impairment in family functioning, leading to an increase in conflict within the family. These findings suggest that continuation of psychostimulant treatment during pregnancy may provide potential benefits for maternal mental health and overall functioning.

However, despite these potential benefits of psychostimulant continuation for maternal mental health, concerns remain regarding fetal and neonatal exposure to these medications, although available data are relatively reassuring. Current evidence suggests that in utero exposure to amphetamines or methylphenidate is not associated with significant increased risk of major congenital or cardiac malformations.^8,9^ Similarly, studies have shown no significant increase in risk of long-term neurodevelopmental disorders in infants, and amphetamine exposure has not been associated with significant differences in infant birth weight, neonatal intensive care unit (NICU) admission, or neonatal abstinence syndrome.^10^ Nevertheless, individualized risk–benefit assessments are necessary to balance the benefits of maintaining maternal mental health with potential fetal and neonatal risks.

In the postpartum period, patients may hesitate to use stimulant or nonstimulant ADHD medications if they plan to breastfeed due to concerns about potential effects on the infant and limited data on specific agents during lactation. However, stimulants like amphetamine and methylphenidate appear to have minimal risk regarding breastfeeding.^11–13^ Bupropion is transmitted through breastmilk in relatively low amounts^14,15^ and is likely compatible with breastfeeding.^16^ Two case reports in the literature reported a seizure in babies exposed to bupropion through breastmilk, though these cases were confounded by infection and multiple medication exposures.^17,18^ Data are limited for clonidine, and adverse effects were reported for one baby exposed to clonidine in utero and during lactation.^19,20^ Data are also limited for atomoxetine, but one recent large cohort study with 990 first trimester exposures did demonstrate reassuring data for use in pregnancy.^21^ Guanfacine currently lacks published safety data.^11^ With limited research on the effects of stimulant use during pregnancy and postpartum management,^8^ this case report explores the outcomes of lisdexamfetamine use during pregnancy and breastfeeding in comparison to untreated ADHD. The reporting of this case conforms to the CARE guidelines (Supplemental Material).^22^

## Case presentation

This patient is a 26-year-old female with a psychiatric history of ADHD, obsessive compulsive disorder (OCD), major depressive disorder, generalized anxiety disorder, and panic attacks. She was initially diagnosed with ADHD along with OCD in elementary school and was able to manage her symptoms through middle school, high school, and college without medications. However, when she continued to struggle with a lack of energy and motivation and when faced with work-related challenges, she began pharmacologic treatment for ADHD. She trialed atomoxetine, which was ineffective prior to starting lisdexamfetamine in 2021. She highlights that “once treated for ADHD, all got better.” In addition, she felt that her symptoms of depression, generalized anxiety, and panic attacks significantly improved or resolved following initiation of lisdexamfetamine.

Further psychiatric history is negative for inpatient admission to mental health unit; psychiatrist and therapy services; prior suicide attempts; self-injurious behavior; past sexual, physical or emotional abuse; and any symptoms suggestive of bipolar disorder, psychotic disorder, or post-traumatic stress disorder. In addition, she denies alcohol use, recreational drug use, marijuana use, and tobacco use.

In her first pregnancy, she experienced obstetric complications of chronic hypertension and preeclampsia with severe features that led to preterm delivery through caesarean section (C-section) at 36 weeks of gestation. She emphasizes that she was not on lisdexamfetamine during her first pregnancy, and her “quality of life was garbage, and going into postpartum was challenging.” Her pregnancy felt like “an emotional rollercoaster the entire time” with extreme emotional dysregulation. In addition, it is important to note that she was not on any other medications during this pregnancy. Her son was born with a cleft palate and micrognathia. Due to preterm delivery and fetal anomalies, her son was admitted to the NICU following birth.

After the birth of her first son, she reports one episode of sustained symptoms of sadness associated with feelings of worthlessness, hopelessness, crying, isolating herself with passive death wishes, irritability, increased sleep, and variable appetite. She mentions that “I would not eat or drink, my entire being was to make sure the baby did not cry and take care of that.” At times, she did not eat unless food was placed in front of her. The first 4 months especially “felt like a cloud where I was fuzzy and disengaged.” In addition, she had no energy or motivation. She took care of the things that she needed to and “nothing more than that.” She highlights that “I knew what I needed to do to make myself feel better but couldn’t get myself to do it.” She was “barely functioning” and could not organize herself enough to decide what to make for dinner, and often would go to bed early.

In her second pregnancy, she also experienced obstetric complications of chronic hypertension that led to preterm delivery via C-section at 33 weeks of gestation. During her pregnancy, her blood pressure remained controlled until around 15 weeks of gestation, at which point she started on labetalol 300 mg. In addition, she was taking aspirin 81 mg and lisdexamfetamine 40 mg daily throughout the pregnancy. She was not on any other medications during this pregnancy. Her repeat C-section was done due to concern for chronic hypertension exacerbation or possible development of superimposed preeclampsia with severe features. While she was inpatient, her blood pressure was uncontrolled despite rapid uptitration of antihypertensive medications. After her son was born, he was admitted to the NICU due to premature birth and congenital anomalies, including cleft palate and micrognathia. She noted that she did not see any differences in her mental health from before she was pregnant to during her pregnancy. She felt stabilized and was “able to get things done.” Her EPDS score during this pregnancy was zero, indicating no signs of depressive symptoms.

Postpartum with her second baby, she reported “feeling better than what I was after the birth of my first baby.” She has not had a “sniff of postpartum depression this time.” She felt good because her ADHD is managed. She “cannot express enough how I am more capable in taking care of myself in this pregnancy and in the postpartum period.” She continued to breastfeed while taking lisdexamfetamine, with no reported concerns to the infant. She states that she had not noticed any side effects in the baby, such as increased jitteriness or sleep changes. In summary, she felt “fantastic.”

It is also important to mention other similarities between two pregnancies (Table 1). In both, she experienced significant third-trimester stressors. In her first pregnancy, she faced work-related stressors, while in her second, she had to mentally prepare for a NICU stay after ultrasounds revealed fetal anomalies similar to those of her first child. Her strongest coping mechanism during both pregnancies included prayers as well as a strong support system from her family and her husband. Written informed consent to publish patient information was obtained from the patient, who had the capacity to provide consent at the time it was obtained and documented by Dr. Ritika Baweja.

**Table 1.** Key characteristics and obstetric outcomes of first pregnancy and second pregnancy.

Key characteristics and outcomes	First pregnancy	Second pregnancy
Maternal age	23 years old	26 years old
Medical/psychiatric history	ADHD, OCD, MDD, GAD, panic attacks	ADHD, OCD, MDD, GAD, Panic attacks
Medication use	None	Lisdexamfetamine 40 mg, labetalol 300 mg, aspirin 81 mg
Obstetric outcome	Chronic hypertension; preeclampsia with severe features	Chronic hypertension; concern for chronic hypertension exacerbation vs development of preeclampsia with severe features
Delivery	Preterm delivery at 36 weeks’ gestation, C-section delivery	Preterm delivery at 33 weeks of gestation, C-section delivery
Neonatal outcome	NICU admission, cleft palate, micrognathia	NICU admission, cleft palate, micrognathia
Breastfeeding	Yes	Yes

ADHD, attention-deficit hyperactivity disorder; GAD, generalized anxiety disorder; MDD, major depressive disorder; NICU, neonatal intensive care unit; OCD, obsessive compulsive disorder.

## Discussion

This patient’s past medical history and social history remained unchanged between her first and second pregnancies. In both, she experienced chronic hypertension, with preeclampsia with severe features in the first and a possible recurrence in the second, which led to preterm delivery in both. However, her mental health between the two pregnancies differed significantly despite comparable coping mechanisms and support systems both times (Table 2). In contrast to her second pregnancy, her first pregnancy and postpartum period were marked by significant challenges in basic daily life functioning. She struggled to meet her fundamental needs, such as eating and drinking, and felt extreme emotional dysregulation. However, while on lisdexamfetamine during her second pregnancy, she noticed a substantial improvement in her mental health, highlighting a shift in her capacity to take care of both herself and her family. She did not experience postpartum depression as she had after her first pregnancy. It is important to note that because the patient was seen during her second pregnancy and outside hospital records were limited, documentation from her first pregnancy was not as comprehensive, and the timing of lisdexamfetamine discontinuation relative to conception in that pregnancy is unknown.

**Table 2.** Qualitative summary of mental health experiences between first and second pregnancy.

Domain	First pregnancy	Second pregnancy
Emotional state (early pregnancy)	“Emotional rollercoaster” “Extremely dysregulated” “Barely functioning”	Stable, no difference in mental health before pregnancy and during. “Able to get things done” “Feels fantastic”
Support system	Married, good support system from family and friends	Married, good support system from family and friends
Coping mechanisms	Praying	Praying
Third-trimester stress	Work-related stressors	Mentally preparing for NICU stay due to similar fetal anomalies seen on ultrasound
Postpartum mental health	“My quality of life was garbage” “Ended up having postpartum depression”	“I am feeling better than what I was after birth of my first baby” “I feel good as my ADHD is managed” “Life is good now” “Feels fantastic”

ADHD, attention-deficit hyperactivity disorder; NICU, neonatal intensive care unit.

Previous studies have suggested a potential association between psychostimulants and preeclampsia. Russell et al.^23^ reported an increased risk of preeclampsia among women exposed to dexamphetamine compared to those who were unexposed. Similarly, Camacho et al.^24^ found an increased risk of preeclampsia in women exposed to psychostimulants compared to those who were unexposed. However, they also report that after adjustments of confounding factors, the association was attenuated, suggesting that psychostimulants are not a major causal factor. Uncertainty around the potential association between psychostimulants and preeclampsia remains. Prior history of preeclampsia has also been studied as a known risk factor for recurrence.^25^ Given this patient’s history of chronic hypertension and preeclampsia despite discontinuation of lisdexamfetamine in her first pregnancy, it is less likely that psychostimulant exposure contributed to the development of chronic hypertension and preeclampsia in her second pregnancy and more likely due to her medical history.

Furthermore, current prospective data on the course of ADHD during pregnancy, demonstrated by an observational cohort study, indicate that women who discontinued psychostimulant treatment during pregnancy had a clinically significant increase in depression and anxiety measured by the EPDS.^7^ Simultaneously, those who discontinued treatment experienced increased family conflict, found parenting more challenging, and felt more isolated. ADHD has been shown to be an independent risk factor for depression and anxiety postpartum,^26^ highlighting the importance of postpartum maternal care. This case study provides an example of the broader literature suggesting that untreated ADHD can contribute to deleterious effects on other aspects of mental health.

Regarding breastfeeding, a recent study observing lactating women taking either lisdexamfetamine or mixed racemic amphetamine salts and their impact on breastfeeding and children’s neurodevelopmental outcomes identified mild adverse effects in 5 out of 13 infants, including somnolence, crying, restlessness, colic, and constipation.^13^ The authors concluded that amphetamine use is likely compatible with breastfeeding. Similarly, this patient reports no concerns with breastfeeding, noting no observed effects on her infant, such as increased jitteriness or sleep changes. Breastfeeding concerns were assessed twice in the postpartum period at approximately 2 and 2.5 months postpartum. Due to the infrequency of the follow-up visits regarding breastfeeding, this limits the ability to fully evaluate for potential delayed effects.

Individualized risk–benefit assessments are fundamental when prescribing stimulants during pregnancy, as they may help determine whether the benefits of treatment of ADHD outweigh the potential risks to both mother and infant. These assessments can factor in the severity of ADHD symptoms, maternal mental health, potential obstetric complications, and fetal outcomes. As seen in this patient, anxiety and mood disorders, including depression, are common comorbidities in women with ADHD and tend to persist into adulthood.^27^ Current literature emphasizes the importance of risk assessment when prescribing medication during pregnancy, as discontinuation may lead to increased daily life impairment, increased depression, and heightened anxiety.^7^ To optimize support and management during the perinatal period, multidisciplinary collaborations involving primary care, obstetrics, and psychiatry may be beneficial for close monitoring, especially for women experiencing more severe symptoms. A multidisciplinary approach between specialties may help optimize support and management during the perinatal period. Although this case report highlights the benefit of continued stimulant use during pregnancy and postpartum, due to the nature that this is a single case report, the findings cannot be generalized. As with all case reports, this is limited by the short follow-up period, inability to establish causality, and potential for incomplete data based on the patient’s recall and availability of records.

## Conclusion

This case report provides one example in which lisdexamfetamine use did not exhibit any severe adverse effects during pregnancy or breastfeeding but instead had a positive effect on mental health. This patient experienced similar pregnancy courses and obstetrics and neonatal outcomes, including chronic hypertension, NICU admission, cleft palate, and micrognathia during both pregnancies, regardless of exposure to lisdexamfetamine. However, she emphasized the difference in her quality of life between her first and second pregnancy, noting the impact of lisdexamfetamine on the treatment of ADHD during pregnancy and the postpartum period. Balancing ADHD symptom management with the potential risks of medications in pregnancy and lactation to maternal and fetal health is challenging. However, the negative impact of untreated ADHD can result in daily-life impairments, occupational impairments, interpersonal impairments, and increased stress.^5,6^ With limited data on stimulants in pregnancy and lactation, there is still a significant need for further research to guide clinical decisions in similar situations.

## Supplemental Material

sj-pdf-1-reh-10.1177_26334941261438664 – Supplemental material for To use or not use lisdexamfetamine in pregnancy and breastfeeding: a case report highlighting ADHD management and maternal–fetal outcomes Supplemental material, sj-pdf-1-reh-10.1177_26334941261438664 for To use or not use lisdexamfetamine in pregnancy and breastfeeding: a case report highlighting ADHD management and maternal–fetal outcomes by Tammy H. Tran, Marissa L. Beal, Melissa F. Free and Ritika Baweja in Therapeutic Advances in Reproductive Health

## Declarations

**Ethics approval and consent to participate:** Our institution does not require ethical approval for reporting individual cases. Written informed consent to publish patient information was obtained from the patient, who had the capacity to provide consent at the time it was obtained, and documented by Dr. Ritika Baweja.

**Consent for publication:** Written informed consent to publish patient information was obtained from the patient, who had the capacity to provide consent at the time it was obtained, and documented by Dr. Ritika Baweja.

**Availability of data and materials:** Not applicable. This case report does not contain any datasets beyond the patient information presented in the manuscript.
