# Supplementary material for: To use or not use lisdexamfetamine in pregnancy and breastfeeding: a case report highlighting ADHD management and maternal–fetal outcomes
Source: Ther Adv Reprod Health. 2026 Apr 5;20:26334941261438664. doi: 10.1177/26334941261438664 (PMC13051099; doi:10.1177/26334941261438664)
Supplement: sj-pdf-1-reh-10.1177_26334941261438664 – Supplemental material for To use or not use lisdexamfetamine in pregnancy and breastfeeding: a case report highlighting ADHD management and maternal–fetal outcomes [file sj-pdf-1-reh-10.1177_26334941261438664.pdf]

CARE Checklist of information to include when writing a case report

CARE case report guidelines

(cc) BY-NC-ND

Topic	Item	Checklist item description	Reported on Line
Title	1	The diagnosis or intervention of primary focus followed by the words "case report"	1-2
Key Words	2	2 to 5 key words that identify diagnoses or interventions in this case report, including "case report"	56-57
Abstract (no references)	3a	Introduction: What is unique about this case and what does it add to the scientific literature?	52-54
	3b	Main symptoms and/or important clinical findings	45-52
	3c	The main diagnoses, therapeutic interventions, and outcomes	45-52
	3d	Conclusion—What is the main "take-away" lesson(s) from this case?	47-54
Introduction	4	One or two paragraphs summarizing why this case is unique (**may include references**)	110-130
Patient Information	5a	De-identified patient specific information.	133-147
	5b	Primary concerns and symptoms of the patient.	148-186
	5c	Medical, family, and psycho-social history including relevant genetic information	133-147; 187-192
	5d	Relevant past interventions with outcomes	139-142
Clinical Findings	6	Describe significant physical examination (PE) and important clinical findings.	Not applicable
Timeline	7	Historical and current information from this episode of care organized as a timeline	148-186
Diagnostic Assessment	8a	Diagnostic testing (such as PE, laboratory testing, imaging, surveys).	177-179
	8b	Diagnostic challenges (such as access to testing, financial, or cultural)	Not applicable
	8c	Diagnosis (including other diagnoses considered)	133-135
	8d	Prognosis (such as staging in oncology) where applicable	Not applicable
Therapeutic Intervention	9a	Types of therapeutic intervention (such as pharmacologic, surgical, preventive, self-care)	169-171
	9b	Administration of therapeutic intervention (such as dosage, strength, duration)	169-171
	9c	Changes in therapeutic intervention (with rationale)	148-171
Follow-up and Outcomes	10a	Clinician and patient-assessed outcomes (if available)	148-186
	10b	Important follow-up diagnostic and other test results	Not applicable
	10c	Intervention adherence and tolerability (How was this assessed?)	170-186
	10d	Adverse and unanticipated events	183-186
Discussion	11a	A scientific discussion of the strengths AND limitations associated with this case report	249-253
	11b	Discussion of the relevant medical literature **with references**.	210-233
	11c	The scientific rationale for any conclusions (including assessment of possible causes)	199-209; 218-226
	11d	The primary "take-away" lessons of this case report (without references) in a one paragraph conclusion	256-267
Patient Perspective	12	The patient should share their perspective in one to two paragraphs on the treatment(s) they received	148-165; 176-186
Informed Consent	13	Did the patient give informed consent? Please provide if requested	Yes [x] No [ ]
